# Prevalence and factors associated with gestational diabetes mellitus in Malaysia: a population-based study comparing 2016 and 2022

**DOI:** 10.1186/s12889-024-20215-3

**Published:** 2024-10-04

**Authors:** Siti Hafizah Zulkiply, Kishwen Kanna Yoga Ratnam, Siaw Hun Liew

**Affiliations:** Institute for Public Health, National Institutes of Health, Ministry of Health Malaysia, Setia Alam, Selangor Malaysia

**Keywords:** Gestational diabetes mellitus, Malaysia, Asia

## Abstract

**Background:**

Gestational diabetes mellitus (GDM) poses substantial health risks to both mothers and infants. Malaysia exhibits a heightened prevalence of GDM.

**Objective:**

This study aims to examine the changes in the prevalence of GDM between 2016 and 2022 and its determining factors.

**Methods:**

The data analysed in this study were derived from the National Health and Morbidity Survey (NHMS) 2016 and 2022, a nationwide study employing a two-stage stratified random sampling design in Malaysia. Changes in the prevalence were compared between data from NHMS 2016 and 2022, while factors were evaluated based on data from NHMS 2022. Descriptive statistics and multiple logistic regression analyses were performed using IBM SPSS version 27.

**Results:**

The prevalence of GDM increased from 12.5% in 2016 to 27.1% in 2022. In both years, the prevalence was highest among those aged 44–49 years, those of Indian ethnicity, those in higher income groups and those with higher education levels. Advanced maternal age, high body mass index (BMI) and hypertension were associated with a greater risk of GDM.

**Conclusion:**

The prevalence of GDM among the Malaysian population doubled from 2016 to 2022. The findings underscore the importance of implementing targeted programs for expectant mothers in high-risk groups to mitigate the incidence of GDM and its associated morbidities.

## Introduction

Gestational diabetes mellitus (GDM) is defined as any degree of dysglycemia that occurs for the first time or is first detected during pregnancy. GDM is one of the leading causes of morbidity for both mothers and infants and has become a global public health burden. GDM is associated with preeclampsia and pregnancy-induced hypertension [1]. In the long term, GDM is associated with an increased risk of developing type 2 diabetes [2–4]. The neonates born to mothers with GDM are associated with macrosomia and respiratory distress syndrome [5]. In addition, they also have an increased risk of childhood and adulthood obesity and an increased cardiometabolic risk [6].

The pooled prevalence of GDM in Eastern and Southeastern Asia was reported to be 10.1% [7]. A similar result (11.5%) was reported in another review of the prevalence of GDM in Asia [8]. The same study reported that the prevalence of GDM in lower or upper-middle-income countries was approximately 64% higher than that in high-income countries [7]. This is supported by another review that reported an overall GDM prevalence of 5.4% in Europe [9]. It has been reported that Malaysia has one of the highest prevalence rates of GDM in Southeast Asia [10]. The reported prevalence of GDM in Malaysia is between 21.5% and 27.9% [11, 12].

Owing to high morbidity, healthcare policymakers need to understand the burden of GDM for further interventions. A nationwide community survey, the National Health and Morbidity Survey (NHMS) has been undertaken periodically in Malaysia to assess the nation’s health. Among others, maternal and child health is also one of the objectives of the NHMS. In two independent studies among the Malaysian population, the prevalence of GDM was reported to be 30%. However, owing to the heterogeneity in the study population, it is difficult to understand the trend of GDM. The present study aimed to evaluate the prevalence of GDM and its associated factors in the Malaysian population between 2016 and 2022.

## Methodology

The NHMS is a nationwide population survey implemented over a 4-year cycle. The Maternal and Child Health survey was conducted in 2016 and 2022. The study population for maternal health included mothers whose last child was under two years old and who were living in non-institutional quarters in Malaysia.

The survey’s sampling frame is derived from the National Population and Housing Census, which divides Malaysia into enumeration blocks (EBs) with several living quarters (LQs), encompassing an average population ranging from 500 to 600. To identify eligible LQs with children aged 0–59 months, listing activities were conducted following the random selection of EBs by the Department of Statistics Malaysia (DOSM). Further details on the study’s design and sampling methodology can be found elsewhere [13, 14].

### Study instrument

The data were collected using a validated structured questionnaire administered via face-to-face interviews with a mobile device.

### Study variables

#### Sociodemographic

Ethnicity was classified based on the three major ethnic groups in Malaysia: Malay, Chinese and Indian. Other categories include ‘Other Bumiputera’ and ‘Other’. ‘Other Bumiputera’ consists of indigenous groups and local populations from Sabah and Sarawak, whereas ‘Other’ includes foreigners and immigrants residing in Malaysia.

The level of education was classified as none, primary, secondary, or tertiary. None referred to having no formal education whereas primary education referred to having completed up to six years of formal schooling. Secondary education refers to 11 years of formal schooling, while tertiary education refers to a diploma or higher qualifications.

#### Gestational diabetes mellitus (GDM)

In Malaysia, screening for GDM is conducted based on risk factors using a 75-gram oral glucose tolerance test (OGTT) which is performed at the time of booking. If the test is negative, it is repeated at 24–28 weeks of gestation. GDM is diagnosed if the fasting plasma glucose (FPG) is > 5.1 mmol/L or if the 2-hour post-prandial (2-HPP) is > 7.8 mmol/L. The participants were asked ‘Have you ever been told by your doctor that you had gestational diabetes mellitus during your pregnancy (with the child that you had the last two years)?

#### Body mass index (BMI)

The participants’ weights for the first 3 months of pregnancy were recorded. If antenatal records were unavailable, their pre-pregnancy weight was recorded instead. Body mass index (BMI) was classified according to the WHO classification, with BMI < 18.5 as underweight, 18.5–24.9 as normal weight, 25-29.9 as overweight and > 30 as obese.

### Statistical analysis

Data from NHMS 2016, and 2022 [13, 14] were analyzed using the complex sample module in the Statistical Package (SPSS) Version 25. Sampling errors were estimated using the primary sampling units and strata provided in the dataset. Sampling weights were used to adjust for non-response bias. As the sampling was a two-stage stratified design, the analysis was done accordingly to ensure that sample weights and design effects were accounted for.

The data was described as a percentage. The overall prevalence was determined for each variable. The increment between surveys was determined by dividing the changes between the later and earlier prevalence with the earlier prevalence and described in percentages.

Multiple logistic regression analysis was conducted to determine the factors associated with GDM. The predictor variables included in the analysis were age, ethnicity, locality, marital status, BMI, locality, household income level, and education level. A p-value of less than 0.05 was considered statistically significant.

## Results

A comparison of the sociodemographic characteristics between populations at NHMS 2016 and 2022, revealed that the percentages of age groups are similar, with most of the participants being between 25 and 34 years old. In terms of ethnicity, the majority of the participants in both groups were Malay. Most participants had a secondary education, lived in urban areas and were married. Table 1 shows the sociodemographic and clinical characteristics of the study participants.

**Table 1:** Sociodemographic and clinical characteristics

Variable	NHMS 2016 (*N* = 10,264) [*n* (%)]	NHMS 2022 (*N* = 7,284) [*n* (%)]
Age group		
15–19	146 (2.8)	75 (1.3)
20–24	1102 (14.5)	643 (10.2)
25–29	2908 (34.0)	1844 (29.0)
30–34	3571 (30.3)	2977 (31.4)
35–39	1889 (14.6)	1302 (20.7)
40–44	586 (3.5)	397 (6.7)
45–49	62 (0.2)	46 (0.8)
Ethnicity		
Malay	7113 (63.2)	4962 (73.5)
Chinese	1128 (14.1)	236 (4.3)
Indians	430 94.5)	207 (3.6)
Other Bumiputera	1181 (12.6)	619 (12.8)
Others	412 (5.7)	282 (5.8)
Marital status		
Single/ Divorcee/ Widower	178 (0.017)	87 (1.4)
Married	10,018 (98.1)	6214 (98.6)
Locality		
Urban	6061 (67.5)	4438 (69.4)
Rural	4203 (32.5)	1920 (30.6)
Education level		
None	78 (0.8)	107 (2.1)
Primary	1072 (11.4)	330 (6.6)
Secondary	5308 (53.6)	3074 (48.3)
Tertiary	3591 (34.2)	2656 (42.9)
Household income level		
< RM 1000	1148 (10.8)	345 (6.3)
RM 1000-RM1999	1619 (15.2)	1684 (24.7)
RM 2000-RM2999	1689 (16.0)	1388 (20.7)
RM 3000-RM3999	1375 (13.1)	973 (14.9)
RM 4000-RM 4999	1030 (10.1)	540 (8.7)
> RM 5000	3391 (34.8)	1414 (24.8)
BMI		
Normal/ Underweight	N/A	3240 (53.1)
Overweight	N/A	1779 (27.6)
Obese	N/A	1148 (19.3)
Hypertension in pregnancy		
Yes	N/A	378 (6.5)
No	N/A	5956 (93.5)
Occupation		
Public sector	2427 (17.5)	921 (14.1)
Private sector	2293 (27.4)	1146 (20.4)
Self-employed	769 (7.2)	499 (7.3)
Housewife/Unemployed/ Student	4677 (47.9)	3609 (58.2)

The prevalence of GDM increased from 12.5% in 2016 to 27.1% in 2022. There is an increase in the prevalence of GDM as age increases, with the highest prevalence among those aged 40 to 44 years old. Among ethnicities, Indians have the highest prevalence of GDM. Table 2 displays the prevalence of GDM and the rate of increase from 2016 to 2022 based on the NHMS data.

**Table 2:** Prevalence of gestational diabetes mellitus

Variable	NHMS 2016 [% (95% CI)]	NHMS 2022 [% (95% CI)]	Rate of increase
	12.5 (11.4,13.6	27.1 (25.6,28.6)	
Age group			
15–19	3.4 (1.3, 8.5)	7.4 (2.2, 22.2)	+ 117.65
20–24	4.7 (3.3, 6.6)	14.4 (11.5,18.0)	+ 206.4
25–29	10.1 (8.8, 11.5)	24.1(21.7, 26.7)	+ 138.6
30–34	13.5 (11.6, 15.6)	28.7(26.2, 31.2)	+ 112.6
35–39	20.5 (18.2, 23.1)	31.7(28.6, 34.9)	+ 54.6
40–44	32.0 (28.1, 36.1)	39.5 (33.5, 45.9)	+ 23.4
45–49	22.2 (13.0,35.1)	35.1 (20.5,53.1)	+ 58.1
Ethnicity			
Malay	13.7 (12.7, 14.7)	28.5 (26.8, 30.2)	+ 108.0
Chinese	9.3 (7.4, 11.8)	22.7 (16.9, 29.8)	+ 144.1
Indians	14.6 (10.3, 20.1)	35.2 (27.5, 43.8)	+ 141.1
Other Bumiputera	11.0 (8.5,14.2)	24.7 (21.1, 28.7)	+ 124.5
Others	8.7 (5.4,13.7)	11.7 (7.3,18.3)	+ 34.5
Marital status			
Single/ Divorcee/ Widower	10.3 (5.6,18.1)	27.2 (25.7,28.7)	+ 164.1
Married	12.5 (11.5,13.7)	12.1 (6.6,21.3)	-3.2
Locality			
Urban	12.5 (11.4, 13.6)	27.6 (25.7,29.6)	+ 120.8
Rural	12.4 (11.0, 14.0)	25.8 (23.6,28.2)	+ 108.1
Education level			
None	10.8 (3.9,26.7)	17.1 (9.9,28.0)	+ 58.3
Primary	10.3 (8.0,13.2)	17.5 (12.6, 23.8)	+ 69.9
Secondary	13.1 (12.0, 14.2)	28.0 (26.0,20.0)	+ 113.7
Tertiary	12.7 (10.7,14.9)	28.1 (25.9,30.4)	+ 121.3
Household income level			
< RM 1000	10.3 (8.3, 12.8)	25.4 (20.9, 30.4)	+ 146.6
RM 1000-RM1999	12.2 (10.3, 14.4)	24.6 (22.1, 27.3)	+ 103.3
RM 2000-RM2999	12.6 910.3, 15.3)	26.5 (23.6, 29.7)	+ 110.3
RM 3000-RM3999	13.4 (11.2, 15.9)	31.0 (27.3, 34.9)	+ 131.3
RM 4000-RM 4999	13.0 (10.2, 16.4)	30.3 (25.0, 36.1)	+ 133.0
> RM5000	12.8 (10.5, 15.4)	27.2 (24.5, 30.0)	+ 112.5
BMI			
Normal /Underweight	N/A	19.9 (18.1,21.8)	
Overweight	N/A	31.9 (29.3,34.6)	
Obese	N/A	41.3 (37.8,45.0)	
Hypertension in pregnancy			
Yes	N/A	53.8 (47.4, 60.0)	
No	N/A	25.2 (23.7, 26.8)	
Occupation			
Public sector	14.8 (12.8,17.1)	27.4 (24.0, 31.1)	+ 85.1
Private sector	12.1 (9.4, 15.5)	24.6 (21.3, 28.1)	+ 103.3
Self-employed	12.1 (9.6,15.0)	25.5 (20.9, 30.8)	+ 110.7
Housewife/ Unemployed/ Student	12.0 (10.6,13.4)	28.2 (26.2, 30.2)	+ 135.0

Table 3 shows the factors associated with GDM. Our study revealed that the significant factors associated with GDM are age group, ethnicity, marital status, hypertension during pregnancy and BMI.

**Table 3:** Factors associated with gestational diabetes Mellitus (NHMS 2022)

Variable	Crude OR [OR (95% CI)]	Adjusted OR [OR (95% CI )]
Age group		
15–19	Ref	Ref
20–24	2.114 (0.571,7.820)	3.999 (1.222, 13.084) *
25–29	3.986 (1.108,14.336) *	5.512 (1.709, 17.778) *
30–34	5.030 (1.402,18.042) *	7.637 92.371, 24.603) *
35–39	5.801 (1.603, 20.996) *	8.717 (2.700, 28.137) **
40–44	8.169 (2.229, 29.940) *	11.515 (3.523, 37.643) **
45–49	6.77 (1.503, 30.548) *	8.783 (2.319, 33.269) *
Ethnicity		
Malays	Ref	Ref
Chinese	0.738 (0.507,1.075) *	0.708 (0.505, 0.993) *
Indians	1.367 (943,1.980) *	1.247 (0.909, 1.717)
Other Bumiputera	0.822 (0.660,1.025)	0.891 (0.726, 1.093)
Others	0.333 (0.196,0.564) **	0.468 (0.326, 0.671) **
BMI		
Normal/ Underweight	Ref	Ref
Overweight	1.887 (2.368,3.409) **	1.830 (1.596,2.098) **
Obese	2.841 (2.368,3.409) **	2.625 (2.259,3.051) **
Hypertension during pregnancy		
Yes	Ref	Ref
No	0.290 (0.221, 0.381) **	0.410 (0.328,0.513) **
Marital status		
Single/ Divorcee/ Widower	Ref	
Married	2.710 (1.378,5.329) *	
Locality		
Urban	Ref	
Rural	0.914 (0.785,1.065)	
Education level		
None	Ref	
Primary	1.024 (0.496,2.114)	
Secondary	1.879 (0.987,3.576)	
Tertiary	1.893 (0.994,3.602)	
Household income level		
< RM 1000	Ref	
RM 1000-RM1999	0.959 (0.715,1.286)	
RM 2000-RM2999	1.061 (0.792,1.420)	
RM 3000-RM3999	1.319 (0.973,1.788)	
RM 4000-RM 4999	1.276 (0.885,1.839)	
> RM5000	1.096 (0.819,1.465)	
Occupation		
Housewife/Unemployed/ Student	Ref	
Public sector	0.961 (0.786,1.174)	
Private sector	0.830 (0.677,1.016)	
Self-employed	0.873 (0.662,1.151)	

## Discussion

This study revealed a twofold increase in the prevalence of GDM rising from 12.5% in 2016 to 27.1% in 2022. These findings are similar to previous studies conducted in Malaysia, which reported a prevalence between 21.5% and 27.9% [11, 12, 15].

This study found that GDM is most prevalent among those aged 35 years and above, those who are obese, single and Indian ethnicity. Although the study’s findings for marital status contradict a previous study, which reported that GDM is more prevalent among married participants [16, 17], caution is warranted in interpreting this finding since the percentages of single, widowed, and divorced participants in this study are very low.

This study identified significant risk factors associated with GDM, including age, ethnicity, BMI, and hypertension during pregnancy. In terms of ethnicity, this study identified a significant risk of GDM linked with Indian ethnicity. This finding is consistent with previous studies in which the rate of GDM was highest among Asian Indians [18, 19]. According to Hedderson et al. (2014), the prevalence rate of diabetes among Asian Indians was 11.1%. A study of phenotypic and genotypic differences between Indians and Scandinavians with GDM found that GDM risk among Indians can be partially attributed to differences in insulin secretion and action [20].

Age, particularly over 35 years has frequently been cited as an important non-modifiable risk factor for GDM [21, 22]. This study reported a similar finding. A previous study reported that the incidence of GDM was linearly correlated with age [23]. Specifically, it has been reported that for each year of increase in maternal age, there is an 8% increase in the risk of GDM [24]. In a separate study, a heightened dose-response relationship was observed among Asian women, indicating a 12.74% increase in risk for each additional year. Furthermore, a study revealed that starting at the age of 25, Asian women had a significantly higher risk of developing GDM than European women did [25]. This may be attributed to metabolic disturbances caused by insulin resistance in GDM, which are closely related to the ageing process. As the body’s intracellular responses to insulin progressively decline with age, this often results in glucose intolerance.

In addition to age, high BMI is another significant factor associated with GDM [26, 27]. This study reported similar findings, where overweight (BMI > 25) and obesity (BMI > 30) were identified as significant risk factors for GDM. A systematic review of GDM risk based on pre-pregnancy BMI found that individuals who are overweight or obese have a 23% higher risk of developing GDM [28]. This is likely due to a high BMI being associated with the clustering of metabolic risk factors for GDM. This includes high fasting plasma glucose, high HbA1c, insulin resistance, high plasma triglycerides and elevated blood pressure [29].

Another modifiable risk factor significantly associated with GDM is hypertension during pregnancy [30]. In another study, a previous history of hypertension during pregnancies was found to be associated with GDM [17, 31]. This study reported a similar finding. Those with hypertension have excessive arterial stiffness and impaired vasorelaxation which could contribute to the worsening of insulin resistance [32].

This study revealed no significant difference between locality, education level, or household income and the risk of GDM. This contradicts previous findings that reported a higher risk of GDM among those in the least quintile [22]. Another study found that women living in urban areas were more likely to develop GDM [33].

### Strength and limitation

The strength of this study lies in its nationwide scope, making it representative of the Malaysian population. In addition, as both surveys share a similar sampling design, they allow for reliable comparisons.

The limitation of this study is that the data on GDM are self-reported, given that the population consists of mothers with children aged 2 years and younger. Another limitation of this study is the limited number of variables. Other risk factors for GDM such as a previous history of GDM, and a family history of GDM, were not included. Additionally, the cross-sectional study design prevents the establishment of a temporal relationship between the factors.

## Conclusion

There is a notable increase in the prevalence of GDM, from 12.5% in 2016 to 27.1% in 2022. The factors associated with GDM among the Malaysian population include older age, high BMI and hypertension during pregnancy.

## Data Availability

No datasets were generated or analysed during the current study.
